# Suicide following treatment with electroconvulsive therapy: A nationwide study of risk factors among 11,780 patients

**DOI:** 10.1192/j.eurpsy.2023.578

**Published:** 2023-07-19

**Authors:** S. D. Østergaard, A. Spanggård, C. Rohde

**Affiliations:** ^1^Aarhus University Hospital - Psychiatry, Aarhus, Denmark; ^2^Aarhus University Hospital - Psychiatry, Aarhus

## Abstract

**Introduction:**

Despite the well-established anti-suicidal effect of electroconvulsive therapy (ECT), patients receiving ECT remain at high risk of dying from suicide.

**Objectives:**

In the present study, we aimed to quantify this risk and identify risk factors for suicide among patients receiving ECT.

**Methods:**

We used nationwide Danish registers to identify all patients that initiated ECT between 2006 and 2016. These patients were matched on sex and age to 10 reference individuals from the general Danish population. First, we compared 2-year suicide risk between patients initiating ECT and the matched reference individuals. Second, we investigated if any patient characteristics were associated with suicide following ECT via Cox proportional-hazards regression.

**Results:**

A total of 11,780 patients receiving ECT and 117,800 reference individuals were included in the analyses. Among the patients receiving ECT, 161 (1.4%) died from suicide within two years. Compared to the reference individuals, patients receiving ECT had a substantially elevated suicide rate (Hazard rate ratio (HRR)=44.5, 95%CI=31.1-63.6). Among those receiving ECT, we identified the following risk factors for suicide: Male sex (HRR=2.3, 95%CI=1.7-3.1), age 60-70 years (HRR=1.6, 95%CI=1.0-2.6), Medium-term higher education (HRR=1.5, 95%CI=1.0-2.2); Long-term higher education (HRR=1.9, 95%CI=1.1-3.1), history of substance use disorder (HRR=2.0, 95%CI=1.4-2.8) and history of intentional self-harm/suicide attempt (HRR=4.0, 95%CI=2.8-5.8).

**Conclusions:**

Among patients receiving ECT, those who are male, aged 60-70 years, have mediumterm to long-term higher education, or have a history of substance use disorder or intentional self-harm/suicide attempt, are at particularly elevated risk of suicide. These findings may guide initiatives to reduce the risk of suicide.

**Disclosure of Interest:**

None Declared

